# Aft2, a Novel Transcription Regulator, Is Required for Iron Metabolism, Oxidative Stress, Surface Adhesion and Hyphal Development in *Candida albicans*

**DOI:** 10.1371/journal.pone.0062367

**Published:** 2013-04-23

**Authors:** Ning Xu, Xinxin Cheng, Qilin Yu, Kefan Qian, Xiaohui Ding, Ruming Liu, Biao Zhang, Laijun Xing, Mingchun Li

**Affiliations:** 1 Key Laboratory of Molecular Microbiology and Technology for Ministry of Education, College of Life Sciences, Nankai University, Tianjin, China; 2 Tianjin University of Traditional Chinese Medicine, Tianjin, China; Louisiana State University, United States of America

## Abstract

Morphological transition and iron metabolism are closely relevant to *Candida albicans* pathogenicity and virulence. In our previous study, we demonstrated that *C. albicans* Aft2 plays an important role in ferric reductase activity and virulence. Here, we further explored the roles of *C. albicans* Aft2 in numerous cellular processes. We found that *C. albicans* Aft2 exhibited an important role in iron metabolism through bi-directional regulation effects on iron-regulon expression. Deletion of *AFT2* reduced cellular iron accumulation under iron-deficient conditions. Furthermore, both reactive oxygen species (ROS) generation and superoxide dismutase (SOD) activity were remarkably increased in the *aft2Δ/Δ* mutant, which were thought to be responsible for the defective responses to oxidative stress. However, we found that over-expression of *C. albicans AFT2* under the regulation of the strong *PGK1* promoter could not effectively rescue *Saccharomyces cerevisiae aft1Δ* mutant defects in some cellular processes, such as cell-wall assembly, ion homeostasis and alkaline resistance, suggesting a possibility that *C. albicans* Aft2 weakened its functional role of regulating some cellular metabolism during the evolutionary process. Interestingly, deletion of *AFT2* in *C. albicans* increased cell surface hydrophobicity, cell flocculation and the ability of adhesion to polystyrene surfaces. In addition, our results also revealed that *C. albicans* Aft2 played a dual role in regulating hypha-specific genes under solid and liquid hyphal inducing conditions. Deletion of *AFT2* caused an impaired invasive growth in solid medium, but an increased filamentous aggregation and growth in liquid conditions. Moreover, iron deficiency and environmental cues induced nuclear import of Aft2, providing additional evidence for the roles of Aft2 in transcriptional regulation.

## Introduction

*Candida albicans,* a common opportunistic human fungal pathogen, can cause superficial mucosal infection as well as life-threatening system diseases in immunocompromised individuals, such as organ transplant recipients, cancer patients and people with HIV/AIDS. In the last few years, *C. albicans* infections occur more frequently with high mortality rates, and are considered as the major sources causing hospital-acquired fungal diseases. Therefore, a better understanding of *C. albicans* pathogenicity will be beneficial to the identification of new antifungal targets and the treatment of *C. albicans* infections. Numerous studies have reported that the pathogenicity of *C. albicans* is relevant to its feature of morphological transition, oxidative stress, as well as iron acquisition and metabolism [1], [2].

Iron is an essential nutrient, which is required for the growth and metabolism in most organisms, including the budding yeast *Saccharomyces cerevisiae* and human fungal pathogen *C. albicans* [3], [4]. Iron cofactors, such as heme and iron-sulfur clusters, are implicated in many major cellular processes, including the tricarboxylic acid cycle, chromatin remodeling and metabolite biosynthesis [4], [5]. However, excess iron is potentially toxic because of the formation of highly toxic radicals by the Fenton reaction [3], [6], [7]. Therefore, it poses a ubiquitous challenge for the organisms to thrive in iron fluctuating environments. It has been widely reported that *S. cerevisiae* is able to employ transcriptional and metabolic remodeling in response to iron fluctuations [4], [8], [9], [10]. The Aft1-Aft2 dependent regulation plays a central role in maintaining iron homeostasis [9], [11], [12]. Aft1 and Aft2 are functionally similar, and have partially overlapping roles in the control of iron-regulated pathways [13], [14]. In iron deficiency, Aft1 activates numerous iron-regulon genes involved in iron binding/acquisition from the environment, the mobilization of intracellular iron stores, and the metabolic adjustments from iron-dependent to iron-independent pathways. Aft2 regulates the transcription of genes involved in intracellular iron homeostasis in the absence of Aft1, including the vacuolar iron transporter *SMF3* and the mitochondrial iron transporter *MRS4*. In addition, previous studies suggested that Aft1 shuttles from cytoplasm into nucleus under iron-deficient conditions, which is crucial for the Aft1 regulation in response to iron availability [15], [16]. In the past several years, numerous attempts have been made to elaborate iron homeostasis regulation in *C. albicans* [17], [18], [19], [20]. To acquire iron from the host environment, *C. albicans* has evolved at least three independent systems, including reductive iron uptake pathway, siderophore-iron uptake pathway and hemoglobin-iron uptake pathway. Moreover, Chen et al dissected an iron homeostasis regulatory circuit among transcriptional activator Sef1 and two other transcriptional repressors (Sfu1 and Hap43), providing a novel insight into the molecular mechanisms of iron metabolism [18]. However, little is known about the role of *C. albicans* Aft-type transcription factor in iron metabolism.

Functional genomics analyses and phenotypic screening experiments reveal that fungal Aft-type transcription factor is implicated in the diverse range of cellular metabolism in *S. cerevisiae*, including iron homeostasis, the RIM101 pH pathway, cell-wall stability, DNA damage, chromosome stability, and mitochondrial function [21]. Notably, the roles of Aft1 in some cellular processes are mediated through an iron-independent mechanism. In addition, a previous report suggested that Aft1 and Aft2 have redundant roles in response to oxidative stress [13]. The *aft1Δ* mutant, particularly the *aft1Δaft2Δ* double mutant, shows hypersensitivity to hydrogen peroxide (H_2_O_2_). Further research revealed that oxidative stress is implicated in the stability of Aft1 regulon mRNAs, and causes metabolic adjustment from the reductive to the non-reductive iron uptake pathways to minimize oxidative damage by the ferrous ions [22].

*C. albicans* has the ability to undergo reversible morphogenetic transitions between budding yeast form and filamentous form. The reversible transition is important for colonization, survival and the establishment of infections in the hostile environment, which is closely associated with pathogenesis and virulence [2], [23]. Multiple environmental sensing and signal transduction pathways involved in morphogenesis and pathogenesis have been extensively characterized in *C. albicans* [24], including Cph1-mediated MAPK pathway, Efg1-mediated cAMP pathway, Rim101-mediated pH response pathway and Czf1-mediated matrix embedding pathway. Some negative regulation pathways are mainly mediated by Tup1 repression with Nrg1, Mig1, and Rfg1 [25], [26]. Cell-cell adhesion (flocculation) and cell-surface hydrophobic interactions are indicative of adhesion properties, which are important for the penetration into internal organs/tissues and establishment of indigenous microbial flora. Many yeast cells, including *C. albicans*, possess the ability to adhere to abiotic surfaces, other cells and host tissues [27], [28], [29]. Adherence is often found to be highly regulated by many transcription factors, such as Bcr1, Als1 and Zap1 [30]. Several signaling cascades are shown to control the adhesion regulation, including the Ras-cAMP-PKA and MAPK-dependent pathways [27].

Our previous study identified a *C. albicans* Aft-type functional homologue *C. albicans* Aft2, an ortholog of *S. cerevisiae* Aft1/Aft2 regulators, and demonstrated its important role in ferric reductase activity and virulence [31]. In this study, we further elucidated the mechanism by which *C. albicans* Aft2 regulated iron acquisition and utilization. Our results suggested that *C. albicans* Aft2 functioned as both a positive and a negative transcription factor in the regulation of different iron-responsive genes. In addition, we also found that the *aft2Δ/Δ* mutant exhibited hypersensitivity to oxidative stress. Deletion of *AFT2* in *C. albicans* increased adherence ability to polystyrene, cell surface hydrophobicity and flocculation. Here, we firstly demonstrated that *C. albicans* Aft2 functioned as a transcription repressor in morphogenesis through regulating the expression of hypha-specific genes in liquid inducing conditions. Furthermore, we provided the direct evidence that iron deficiency and environmental cues induced nuclear localization of Aft2, which was usually a prerequisite for transcriptional control.

## Materials and Methods

### Strains and Growth Conditions

All strains used in this study are listed in Table 1. Strains were routinely cultivated in YPD medium (1% yeast extract, 2% peptone, 2% glucose) supplemented with 80 µg/ml uridine or synthetic drop-out medium (0.67% yeast nitrogen base without amino acids, 2% glucose, 0.2% complete amino acid mixture lacking specific amino acids). For dot assay experiments under various conditions, YPD medium containing calcofluor white (CFW), sodium dodecyl sulfate (SDS), Na^+^, Zn^2+^, Cd^2+^ and Co^2+^ was achieved, respectively. YPD medium supplemented with 10% fetal bovine serum and Spider medium were used for hyphal induction. 100 µM bathophenanthroline disulfonate (BPS, Sigma) was added to YPD or M199 medium (Gibco) to achieve iron-deficient conditions. For oxidative stress assay, hydrogen peroxide (H_2_O_2_) at the indicated concentrations was added to YPD medium.

**Table 1:** Strains and plasmids used in this study.

Strain and plasmid	Genotype	Source
*C. albicans*		
BWP17	*ura3::λimm434/ura3::λimm434 his1::hisG/his1::hisG arg4::hisG/arg4::hisG*	D. David
NKF23	BWP17 *aft2::ARG4/AFT2*	31
NKF25	BWP17 *aft2::ARG4/aft2::URA3-dpl200*	31
NKF46	BWP17 *aft2::ARG4/aft2::dpl200,* pCR4*-AFT2*	31
NKF47	BWP17 *aft2::ARG4/aft2::dpl200,* pCR4	31
NKF55	BWP17 *aft2::ARG4/AFT2-HA*	This study
NKF58	BWP17 *RP10:: P_ACT1_-AFT2-GFP-URA3*	This study
NKF90	BWP17 *aft2::ARG4/aft2::dpl200,* pBES116*-P_ADH1_*	This study
NKF91	BWP17 *aft2::ARG4/aft2::dpl200,* pBES116*-P_ADH1_-AFT2*	This study
*S. cerevisiae*		
W303a	*MATa leu2-3,112 ura3-1 trp1-92 his3-11,15 ade2-1 can1-100*	31
NKF24	W303a YEplac195	31
NKF50	W303a *aft1::TRP1*	31
NKF52	W303a *aft1::TRP1,* YEplac195	31
NKF53	W303a *aft1::TRP1,* YEplac195*-*Ca*AFT2*	31
NKF55	W303a *aft1::TRP1,* YEplac195*-P_PGK1_-*Ca*AFT2*	31
Plasmids		
pDDB211	containing *LacZ* marker, Amp^r^	32
pDDB78	containing *TRP1* marker, Amp^r^	32
pNKFrp1	the *HIS1* vector with *C. albicans FRP1*promoter and *LacZ* gene	This study
pNKSit1	the *HIS1* vector with *C. albicans SIT1* promoter and *LacZ* gene	This study
pBES116	*ADE2-URA3-ADE2*, *Asc*I fragment in pBluescript II KS (+)	G. Fink
pBES116*-P_ADH1_*	∼1.8 kb *ADH1* promoter in pBES116	This study
pBES116-*P*_ADH1_-*AFT2*	∼2.4 kb *C. albicans AFT2-*ORF and ∼0.56 kb terminator in pBES116*-P_ADH1_*	This study
pFA-HA-URA3	HA tagging vector	This study
pFLAG-ACT1	the FLAG tagging *C. albicans ACT1* promoter vector	This study
pFLAG-GFP	∼0.8 kb *C. albicans GFP*-ORF in pFLAG-*ACT1*	This study
pFLAG-*AFT2*-GFP	∼2.4 kb *C. albicans AFT2*-ORF in pFLAG-*GFP*	This study

### Strains and Plasmids Construction

Primers used in this study are listed in Table 2. The pNKSit1 promoter fusion, which included the native *CaSIT1* promoter and LacZ reporter, was generated as described previously [32]. Briefly, a ∼1 kb of *SIT1* promoter was amplified from the BWP17 genome (primers 1/2), and ligated into pGEM-Teasy vector (Promega) to generate pGEM-T-*P_SIT1_*. Then, a ∼3.5 kb *Ase*I-*Mlu*I fragment containing the *LacZ* gene digested from pDDB211 plasmid was cloned into the *Nde*I-*Mlu*I sites of pGEM-T-*P_SIT1_* to yield pGEM-*P_SIT1_*-LacZ. Both a ∼5.5 kb *Drd*I fragment digested from pGEM-*P_SIT1_*-LacZ and a ∼7.3 kb *Not*I-*Eco*RI product containing *TRP1* marker digested from pDDB78 plasmid were transformed into a *trpΔ S. cerevisiae* by in vivo recombination to generate pNKSit1 plasmid, which was recovered into *Escherichia coli* by electroporation. The pNKFrp1 plasmid was constructed by the same strategy (primers 3/4). The resulting plasmid was digested with *Nru*I and transformed into *C. albicans* strains for β-galactosidase assays.

**Table 2:** Primers used in this study.

Primer	Sequences (5'-3')^a^
1	AATGACCCATTTCTTGCCATTG
2	CATATG GCTAGCAAAAAAAAAAACGG
3	ACGTTTATACAAGAAGGTAC
4	CATATG TGAAAGTTAAACTTGGTT
5	GCAAGATCTTCCATGACTGATAGAGTCACTCATCGAG
6	ACGCGTCGA**C**TGCGATGAAGCTATTGGTGTCACAGG
7	GCGCGGATCCCATGACTGATA GAGTCACTCATC
8	CCGCTCGAGCCAACCAGGTCCATAATTG
9	AGTGGTAATACAAGTAATAATTTATTGAATGATATGCCCAATTATGGACCTGGTTGGCCCGGGTACCCATACGATGTTC
10	TAACAAACTATAAATTAAATAAATATAAAATTAACACACGAAGAAATGAAATGAAGTTGATACGACTCACTATAGGGAG
11	TGTCTACACTACATTCTGTC
12	AGGAATAGATGGTTGTGAAC
13	GTTCTAACCCAGGTGCTG
14	TCCAGAACCAGAGCCATC
15	CCAAGCACCTACTGTTCC
16	GATACCAGCAACAACAGAAT
17	CTCATTACACCAACCATACA
18	GGATTCTGTGGTTGTAGTAT
19	GGATTCTACGAGTACTCATC
20	TGACATGCCTAAAATTCTGG
21	CTGGTGCTGTGTTGTTCC
22	AGCCCACATCCCGATACC
23	GAAGGGTTTATCAATTGGAC
24	ATGGTGCACTGACAATTGGT
25	GATCACCACCTTCATCATCTG
26	GACGCTGGAGTATCAACACT
27	CTTCATCGTTTTCAGAGAAT
28	CAACCAATAATAAGACAGAC
29	TTCCACGGTTTATTCCAGCAC
30	GACACCTCTCATACCATCAC
31	GAGTGGTTGATATTTGTATT
32	GTAGAAATCGCCCCATACATG
33	AAGTGGTAAAGGCAACAGCG
34	ACCAAGTAACCCTGAACCGT
35	CATACTTTTATACCTGTAACC
36	TCGATTATCAAAGGCCAAAC
37	ATTATTTGCTGCCATCACGTTG
38	GCTTTATCAGTGGTTTCGTTGA
39	TGATTTGTGGACCGTAGCTGA
40	GCCAACACAGCAGCATTTACA

a Restriction sites are underlined.

A ∼3.0 kb *Bgl*II/*Sal*I PCR fragment (primers 5/6) was subcloned into the pBES116*-P_ADH1_* plasmid to yield the *AFT2* complemented construction under the strong *ADH1* promoter. A ∼2.4 kb *Bam*HI/*Xho*I *AFT2* ORF product (primers 7/8) was subcloned into pFLAG-GFP to yield the Aft2-GFP fusion plasmid. *Stu*I-linearized fusion plasmid was integrated into the *RP10* locus to express the GFP fusion protein under the *ACT1* promoter in *C. albicans.* All the constructs were verified by DNA sequencing. The Aft2-HA fusion protein was achieved by PCR-mediated homologous recombination. The *aft2::URA3* cassette containing a flanking homology region was amplified according to the 9/10 primers and the pFA-HA-URA3 template. Then, the *AFT2* heterozygous mutant was transformed by the *aft2::URA3* cassette to generate correct Aft2-HA recombinants.

### RNA Isolation and Quantitative Real-time PCR

Total RNA was extracted by the phenol-chloroform method as previously described [19]. The overall quality of RNA was measured by A260/A280 and analyzed by agarose gel electrophoresis. Quantitative real-time PCR was performed in triplicate and repeated in three independent experiments using an iQ5 Real-Time PCR system (Bio-Rad). Primers (11–40) used in quantitative real-time PCR are listed in Table 2. Independent reaction mixtures were carried out by the same cDNA for both the genes of interest and the *ACT1* gene using the SYBR Green qPCR SuperMix (TransGen Biotech) according to the instructions. Thermal cycler conditions included initial denaturation at 95°C for 3 min, followed by 40 cycles of denaturation at 95°C for 20 sec, annealing at 58°C for 20 sec and elongation at 72°C for 20 sec. The relative fold changes in gene expression were determined by the 2^−*△△CT*^ method [33].

### β-galactosidase Assays

Overnight cultures of strains in M199 (pH 4) medium were washed and re-cultivated in 35 ml fresh M199 medium buffered with 150 mM HEPES to pH 4 and pH 8 with or without 100 µM BPS to mid-exponential phase at 30°C. Then, β-galactosidase assays were performed as described previously [32]. The activity was calculated in Miller units according to the following formula: (A420)/(OD_600_×volume assayed×time).

### Reactive Oxygen Species (ROS) and Superoxide Dismutase Activity (SOD) Assay

Determination and detection of ROS were performed as described previously [34], [35]. The indicated strains were cultivated to mid-exponential phase, and stained with 10 μΜ 2', 7'-dichlorofluorescein-diacetate (DCFH-DA) for 30 min. Then, cells were washed two times with phosphate-buffered saline (PBS) buffer, and incubated for 90 min in YPD medium supplemented with 8 mM H_2_O_2._ The fluorescence signals, an indicator of the degree of general oxidative stress, were measured by a Zeiss Axio Imager Z1 fluorescence microscopy.

SOD activity was determined by the Riboflavin/NitroBlue Tetrazolium (RF/NBT) assay as described previously with slight modifications [36], [37]. Superoxide dismutase catalyses the inhibition of NBT reduction, and the extent of which could be monitored by spectrophotometry. Mid-exponential cells were suffered to 8 mM H_2_O_2_ for 90 min, washed for three times and weighted. Crude cell extracts were prepared by breakage with glass beads in 0.1 M ice-cold Na_2_HPO_4_-NaH_2_PO_4_ buffer containing protease inhibitors. After centrifugation, culture supernatants were collected. NBT reduction was measured in five reaction mixtures containing 0 µl, 40 µl,80 µl,120 µl and 300 µl cell extract. The 1 ml reaction mixtures contained 50 mM Na_2_HPO_4_-NaH_2_PO_4_ buffer, 130 mM methionine, 20 µM riboflavin, 750 µM NBT and 1 µM EDTA. The samples were incubated in the dark for 10 min, and subsequently illuminated by 4 fluorescence tubes (Philips TLD/18 W, 4500 lux) for 20 min at the room temperature. Afterwards, absorbance was measured at 560 nm by spectrophotometry. The sample amount of 50% inhibition was calculated by regression using the linear part of a natural semi-log curve, and regarded as one unit of SOD enzyme activity (U). The SOD activity was expressed as U/(g×h) reagent, and calculated by following formula: (volume of assayed sample×1000×60)/(volume of 50% inhibition×weight of assayed sample×reaction time).

### Adhesion, Flocculation and Cell Surface Hydrophobicity

The crystal violet assay was used to quantify adhesion ability [38]. Briefly, Overnight cultures were washed and resuspended to an optical density of 5×10^6^ cells/ml in RPMI 1640 medium (buffered to pH 7.4 with 165 mM morpholinepropanesulfonic acid). Suspensions of each strain (500 µl) were added into a separate 24-well polystyrene plate, and incubated statically at 37°C. After 2 h, 4 h and 12 h incubation respectively, each plate was gently washed with PBS buffer for three times to remove any non-adherent cells. The adherent cells were fixed with 1 ml of methanol for 15 min, stained with 1% crystal violet for 2 min, washed gently and submerged with 10% acetic acid for 15 min. The A595 of the supernatant was measured to determine the biomass of each sample. After 12 h incubation, structure of adhered cells was examined by Scanning Electron Microscopy (SEM) as previously described [39]. Samples were visualized at a magnification of ×2000 by using a Lecia Cambridge S-360 scanning electron microscope.

Cell flocculation assay was examined as follows: Firstly, *C. albicans* strains were grown in YPD medium for 24 h at 37°C. Cells were harvested and resuspended in RPMI 1640 medium with OD_600_ of 0.1, then cultivated to mid-exponential phase for 12 h at 37°C with shaking. The tubes were vortexed for 30 sec and allowed to settle for 10 min before photographed.

Cell surface hydrophobicity was determined as described in the previous study [40]. Cells were cultivated to mid-exponential phase, washed twice with PBS buffer, and resuspended to an OD_600_ of 1.0. For each strain tested, 200 µl aliquots of xylene or hexadecane were added to glass tubes containing 3 ml of cell suspension. After 10 min incubation at 37°C water bath, the mixtures were subjected to a vigorous vortexing. Before the OD_600_ of the aqueous phase was measured, the tubes were allowed to incubate for a further 20 min to separate the hydrocarbon from the aqueous phase. The percentage of cells removed from the aqueous phase was expressed as cell surface hydrophobicity. Each strain was tested in triplicate and two independent situations.

### Fluorescence Microscopy

To observe the Aft2 subcellular localization in response to environmental cues, wild-type strains producing Aft2-GFP fusion protein were cultivated to mid-exponential phase in YPD, YPD+100 µM BPS, YPD+10% serum and Spider medium, respectively. For GFP microscopy, cells were fixed with 3.7% formaldehyde in the culture medium for 30 min, washed, and stained with 1 µg/ml 4',6-diamidino-2-phenylindole (DAPI, Sigma) for 10 min. Cells were washed for three times, examined and photographed with a Leica TCS SP5 confocal laser scanning microscope.

## Results

### *C. albicans* Aft2 Plays an Important Role in Iron Metabolism under Iron-deficient Conditions

**Figure 1:**
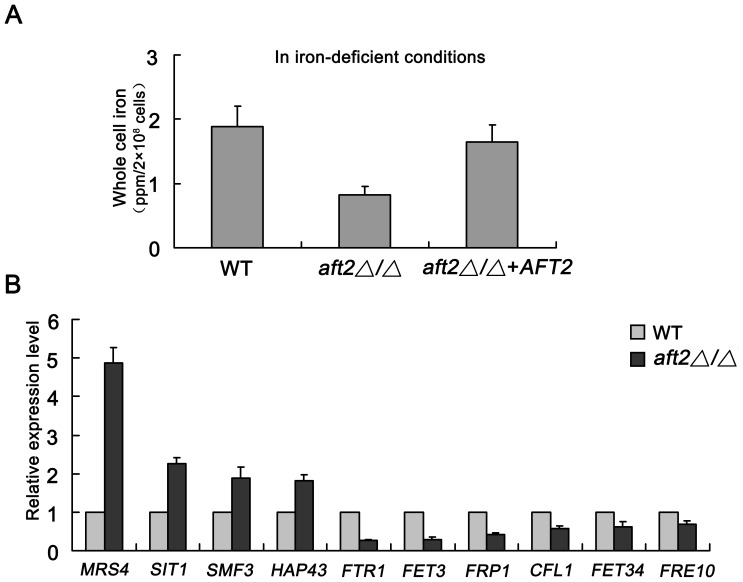
Effect of *AFT2* deletion on iron content and iron-regulon expression under iron-deficient conditions. (A) Cells were pre-grown in YPD medium for 24 h at 30°C, and re-cultivated in 100 ml fresh YPD+100 µM BPS medium for 12 h, respectively. Then, cells were washed twice with sterilized PBS buffer, twice with 1 mM EDTA, and followed by twice with distilled deionized water to remove all exogenous iron. Measurement of cellular iron levels was performed by a Hitachi 180-80 polarized Zeeman atomic absorption spectrometry. Note: ppm =  mg/kg. (B) Overnight cultures of the wild-type and *aft2Δ/Δ* mutant strains were cultivated to mid-exponential phase in YPD+100 µM BPS medium, and used for RNA isolation. Quantitative real-time PCR was performed to determine the relative expression changes of iron-responsive genes. Data indicate mean values ± standard deviations from three independent experiments performed in triplicates.

Our previous study revealed that deletion of *AFT2* affects cell surface ferric reductase activity [31]. To further investigate the role of *AFT2* in iron metabolism, cellular iron content was determined by atomic absorption spectroscopy. Our results demonstrated that deletion of *AFT2* had no significant effect on cellular iron content under iron-adequate conditions (Figure S1A). However, in comparison with the wild-type (BWP17) and *AFT2* complemented (NKF46) strains, cellular iron levels in the *aft2Δ/Δ* mutant (NKF25) were reduced approximately 50% under iron-deficient conditions (Figure 1A). These results indicated that deletion of *AFT2* reduced cellular iron accumulation, suggesting a potential role of *AFT2* in iron metabolism. To explore the mechanism by which *AFT2* affected iron metabolism, quantitative real-time PCR was performed to examine the expression of iron-regulon genes. When grown under iron-adequate conditions, the transcript levels of iron-regulon genes were similar in the wild-type and *aft2Δ/Δ* mutant cells (Figure S1B). However, *C. albicans* Aft2 had a dual role in regulation of iron-dependent genes in response to iron deficiency (Figure 1B). Aft2 acted as a negative regulator to govern the expression of some iron-regulon genes, such as *SIT1*, *MRS4, SMF3* and *HAP43*. On the other hand, it also functioned as a positive regulator to induce the expression of some other iron-related genes, such as *FRP1*, *CFL1*, *FET3*, *FET34* and *FTR1*. Taken together, our results indicated that *C. albicans* Aft2 is closely associated with the regulation of iron-responsive genes under iron-deficient conditions.

### *C. albicans* Aft2 is Involved in Iron-responsive Transcriptional Regulation in Different pH and Iron-limited Conditions

**Figure 2:**
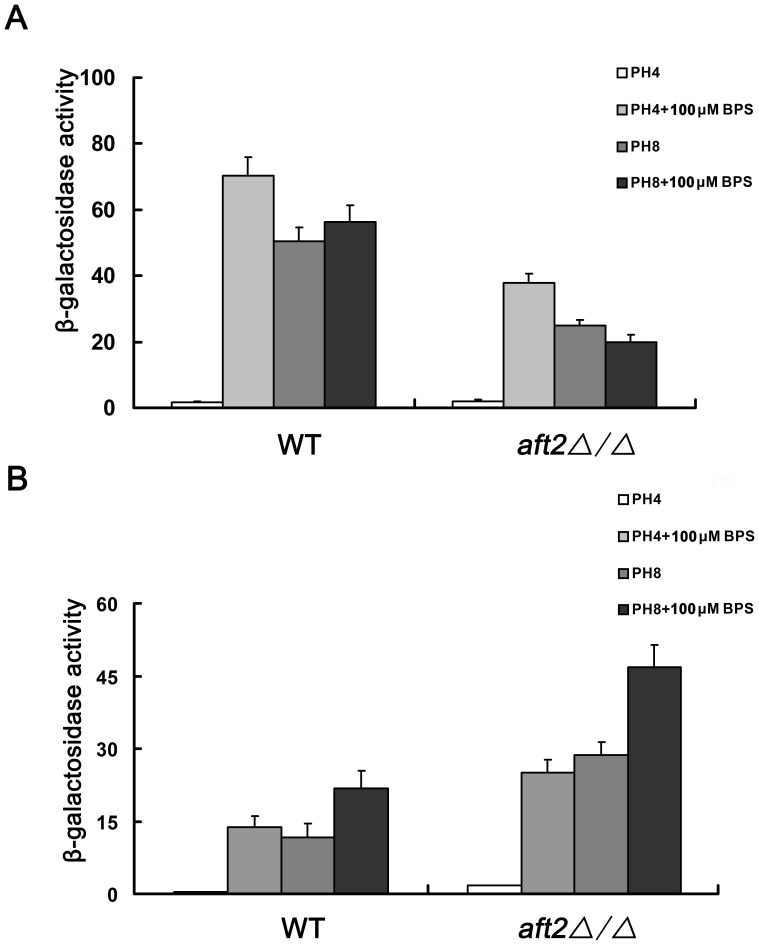
*C.albicans* Aft2 has an effect on the expression of *FRP1* (A) and *SIT1* (B). All strains were grown to mid-exponential phase in M199 medium at pH 4 and pH 8 with or without 100 µM BPS, and β-galactosidase activity was examined as described in Materials and methods. At least two replicates and three independent transformants in each experiment were used to determine average Miller units, and error bars represent the standard deviation of triplicate assays.

To further understand the regulatory mechanism of Aft2 transcription factor, both the *P_FRP1_-*LacZ *(*pNKFrp1) and *P_SIT1_-*LacZ (pNKSit1) promoter fusions were constructed and transformed into the wild-type and *aft2Δ/Δ* mutant strains, respectively. The expression of LacZ reporter, as an index of promoter activity, was examined in different pH and iron-limited conditions. In *aft2Δ/Δ* cells, the *P_FRP1_-*LacZ expression was respectively reduced ∼ 46% at pH 4 with 100 µM BPS, ∼ 51% at pH 8 and ∼ 65% at pH 8 with 100 µM BPS, compared to the results for wild-type cells (Figure 2A). These results demonstrated that *C. albicans* Aft2, as a transcription activator, was related to *FRP1* induction under both alkaline environment and iron deficiency. On the contrary, the *P_SIT1_-*LacZ expression was induced ∼ 1.8-fold at pH 4 with 100 µM BPS, ∼ 2.0-fold at pH 8 and ∼ 2.2-fold at pH 8 with 100 µM BPS, respectively (Figure 2B). These data suggested that *SIT1* repression was also dependent on *C. albicans* Aft2 under both alkaline environment and iron deficiency. Notably, both genes had low expression under acidic condition (pH 4). In acidic pH condition, there was no significant difference of *FRP1* expression between wild-type and *aft2Δ/Δ* cells. However, *SIT1* expression still retained ∼ 4.5-fold induction in *aft2Δ/Δ* cells (Figure 2A, B). These results appeared to raise the question that Aft2 might play no or only a minor role in iron-responsive gene expression, especially in acaid pH condition. Although the present data could not sort out direct and indirect iron-regualtory events, these results at least partially suggested that *C. albicans* Aft2 has an effect on the expression of *FRP1* and *SIT1* under alkaline environment and iron deficiency.

### Deletion of *AFT2* Results in Hypersensitivity to Oxidative Stress

**Figure 3:**
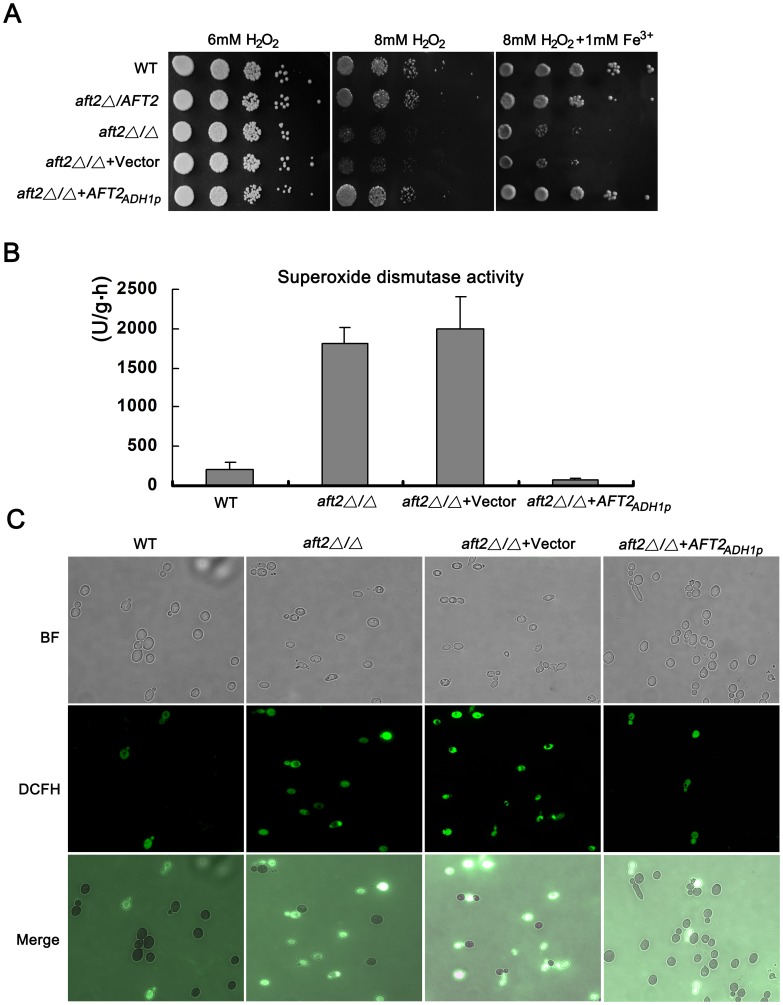
Aft2 is required for the oxidative stress response. (A) Overnight cultures of each strain were resuspended in YPD medium with OD_600_ of 0.1. 5 ul of cells in 10-fold serial dilutions were spotted onto solid YPD or YPD +1 mM Fe^3+^ medium that was supplemented with the indicated concentrations of H_2_O_2_. The plates were incubated at 30°C for 2 days and photographed. (B, C) Reactive oxygen species (ROS) and superoxide dismutase enzymes (SOD) activity were measured as described in Materials and methods.

Phenotypic analyses have indicated that *S. cerevisiae aft1Δ* mutant exhibits several phenotypes related to oxidative stress, including hydrogen peroxide (H_2_O_2_) hypersensitivity and oxygen-dependent copper toxicity [13]. To explore the function of *C. albicans Aft2* in the oxidative stress response, we investigated the sensitivity of the *aft2Δ/Δ* mutant to H_2_O_2_ treatment. There was no significant growth difference between the wild-type and *aft2Δ/Δ* mutant strains at low concentrations of H_2_O_2_ (Figure 3A, left panel). However, when exposed to a higher concentration of H_2_O_2_, deletion of *AFT2* resulted in a severe growth defect in *C. albicans* (Figure 3A, middle panel). The growth defect of the *aft2Δ/Δ* mutant could be rescued by integrating a wild-type *AFT2* fragment under the control of *ADH1* promoter, and the complemented strain showed similar phenotypes in comparison with the *aft2Δ/AFT2* heterozygote and wild-type strains (Figure 3A, middle panel). To explore the possible correlation between Aft2-mediated iron regulation and resistance to oxidative stress, 1 mM exogenous iron was added to the medium. As shown in Figure 3A, exogenous iron could increase growth ability of all strains in response to oxidative stress. However, compared with the phenotype of the wild-type strain, the *aft2Δ/Δ* mutant still showed the hypersensitivity to H_2_O_2_ treatment after the addition of exogenous iron (Figure 3A, right panel). These results suggested that the enhancement of growth is independent of *AFT2* status and the growth defect of the *aft2Δ/Δ* mutant might be mediated in an iron-independent fashion.

To expound the nature of growth defects of the *aft2Δ/Δ* mutant in response to H_2_O_2_ treatment, we measured the levels of reactive oxygen species (ROS) production and superoxide dismutase (SOD) activity. As compared to the complemented and wild-type strains, the *aft2Δ/Δ* mutant exhibited a dramatically elevated level of SOD activity (Figure 3B). Deletion of *AFT2* highly increased generation and accumulation of ROS. Fluorescence assays suggested that ∼72% of the *aft2Δ/Δ* cells harbored high levels of ROS production, whereas ∼18% of wild-type cells had increased ROS accumulation (Figure 3C). These results indicated that *C. albicans AFT2* is closely associated with the oxidative stress response.

Regarding the above conclusion that Aft2 had a role in regulation of iron-regulon genes, further research was performed to evaluate the effect of oxidative stress on the expression of iron-regulon genes in the *aft2Δ/Δ* mutant. Quantitative real-time PCR analyses revealed that, when exposed to oxidative stress, deletion of *AFT2* had no significant effect on the transcript levels of iron-regulon genes (Figure S2). These results demonstrated that the effect of *AFT2* deletion on iron-regulon expression might not be due to secondary effects of the sensitivity to oxidative stress.

### *C. albicans* Aft2 does not Effectively Rescue the Non-iron Related Metabolic Defects of the *S. cerevisiae aft1Δ* Mutant

**Figure 4:**
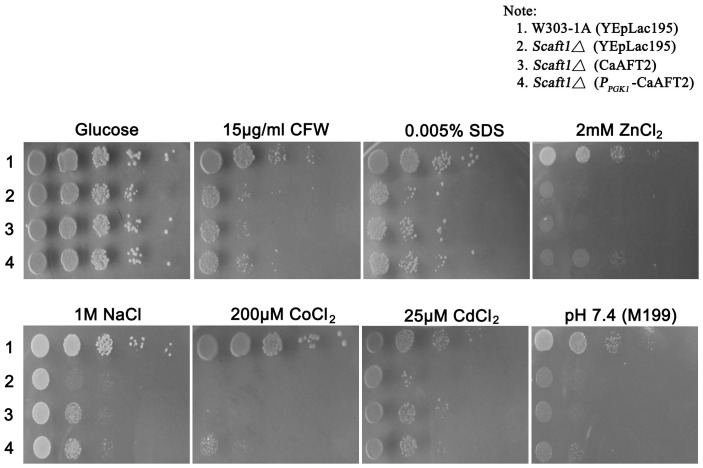
Aft-type regulator in *C.*albicans fails to complement the non-iron related phenotypes of *S. cerevisiae aft1Δ* mutant. Overnight cultures of *S. cerevisiae* W303 derivatives (NKF24, NKF52, NKF53 and NKF55) were grown to mid-exponential phase, and resuspended in YPD medium with OD_600_ of 0.5. 3 µl of cells in 10-fold serial dilutions were spotted onto plates and incubated at 30°C for 2 days.

In our previous study, we confirmed that *C. albicans* Aft2 is able to rescue *S. cerevisiae aft1Δ* mutant growth defects under iron-limited conditions [31]. To further determine whether *C. albicans* Aft2 remained the functional diversity of Aft-type regulator in cellular metabolism, phenotypic analyses of the derivative strains were examined. The results indicated that, under the control of either native or *S. cerevisiae* strong *PGK1* promoter, *C. albicans* Aft2 could not effectively rescue the *Scaft1Δ* defective phenotypes in cell-wall integrity (CFW and SDS), metal ion sensitivity (Zn^2+^, Na^+^, Co^2+^, and Cd^2+^) and alkaline pH resistance (pH 7.5) (Figure 4). Interestingly, there were no significant growth differences between *C. albicans* the wild-type (BWP17) and *aft2Δ/Δ* mutant strains under the same tested conditions, suggesting that *C. albicans* Aft2 might not exert the essential role in the tested metabolic processes (data not shown).

### *Aft2* is Required for Invasive Growth in *C. albicans*

**Figure 5:**
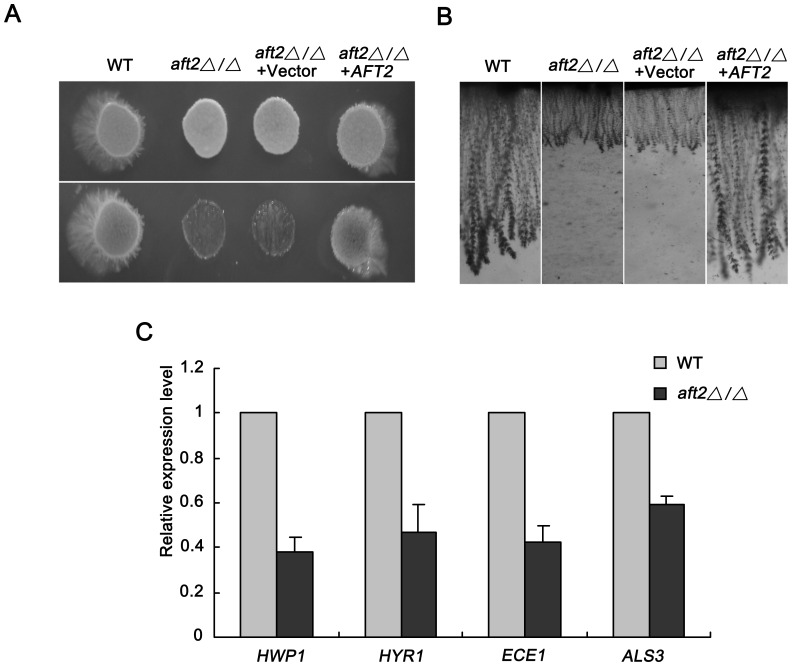
Deletion of *AFT2* affects the ability of invasive growth in *C.albicans*. Wild-type, *aft2Δ/Δ* mutant, *aft2Δ/Δ*+Vector (NKF47) and *AFT2* complemented strains (NKF46) were resuspended to OD_600_ ≈ 0.1. 5 µl suspension was respectively spotted onto solid M199 medium (pH 7.4) and Spider medium. (A) The Spider plates were photographed before and after wash. Analogous results were observed for growth on two different solid medium. (B) The agar medium was cut into 1-mm thick slices and the invasion was examined under the microscope. (C) Cells growing in solid Spider medium for 5 days were harvested by mechanical disruption in cold PBS buffer and used for RNA extraction. Expression analyses of selected hypha-specific genes were assessed by quantitative real-time PCR, and data are representative of three independent experiments.

In the previous study, we found that deletion of *AFT2* affects colony morphology in solid inducing medium [31]. To further investigate the role of *C. albicans* Aft2 in hyphal development, we tested the invasive growth. 5 ul of the indicated strains was spotted on solid M199 and Spider medium, respectively. After 5 days incubation at 37°C, the plates were observed before and after wash. Our results suggested that deletion of *AFT2* severely impaired invasive growth, almost all the mutant cells on the surface were washed off (Figure 5A). On the contrary, a great many wild-type and complemented cells still remained on the surface, suggesting a strong invasive growth. Microscopy-based assay was performed to further determine the degree of invasion (Figure 5B). The wild-type strain exhibited long filaments surrounded by numerous yeast form cells in the septa, which was similar to the observation in the complemented strain. However, the *aft2Δ/Δ* strain showed multiple branches of short and stunted filaments. To understand the nature of invasive growth defect of the *aft2Δ/Δ* mutant, total RNA was extracted to analyze the transcript levels of hypha-specific genes. Surface proteins, as pro-adhesive and pro-invasive factors, are closely related to the morphogenesis and pathogenicity of *C. albicans* [24]. *HWP1* and *HYR1* encode GPI-anchored cell wall proteins, which are required for adherence and phenotype switching [41], [42]. *ECE1* encodes hypha-specific cell wall-linked component, which is in association with the extent of hyphal cell elongation [43]. *ALS3*, as a major member of the *ALS* family, encodes the cell surface glycoprotein. It is known as an essential adhesion and implicated in the adherence to host surfaces and biofilm development [44], [45], [46]. Our data revealed that deletion of *AFT2* reduced the expression of hypha-specific genes in solid inducing conditions, which was consistent with the colony morphology and invasive growth defects in the *aft2Δ/Δ* mutant (Figure 5C). Taken together, these findings suggested that *C. albicans* Aft2 is required for invasive growth.

### *C. albicans* Aft2 Functions as a Repressor in Flocculation, Plastic Adhesion, and Surface Hydrophobicity

**Figure 6:**
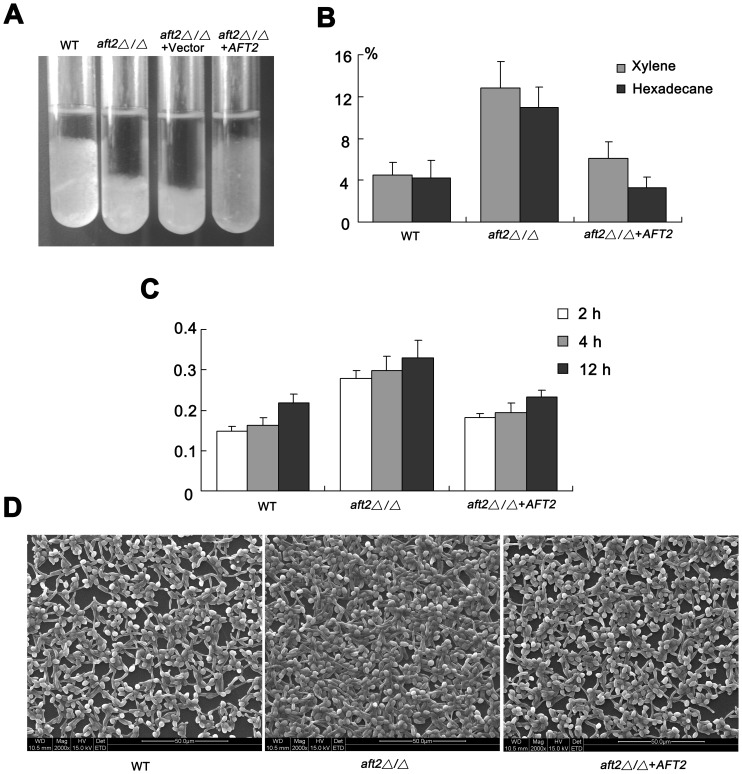
Influence of *AFT2* on surface properties in *C.albicans*. (A) The strains indicated were cultivated in RPMI 1640 medium to mid-exponential phase at 37°C. All tubes were vigorously vortexed, and cells were allowed to settle for 10 min before the flocculation observation. (B) Cell surface hydrophobicity was determined by the hydrocarbon/water partition method with xylene and hexadecane. Data indicate mean values ± standard deviations from three independent experiments performed in triplicates. (C) After 2 h, 4 h and 12 h incubation, the ability of adhesion to polystyrene was assessed through the quantification of total biomass by crystal violet staining. (D) The SEM assay was performed after 12 h incubation in a 24-well cell culture plate.

In view of the above-mentioned result that Aft2 was implicated in agar invasion, we further explored the possible role of Aft2 in surface adhesion properties. Cell flocculation ability was measured after growth in RPMI 1640 medium (Figure 6A). Deletion of *AFT2* resulted in a small but significant increase in flocculation. However, ectopically expression of *C. albicans* Aft2 suppressed the flocculent phenotype of the *aft2Δ/Δ* mutant. These results indicated that Aft2 acted as a repressor in cell flocculation. In addition, cell surface hydrophobicity was also evaluated by the hydrocarbon partition test. Consonantly, the *aft2Δ/Δ* strain showed a higher cell surface hydrophobicity in comparison with the results for the wild-type strain (Figure 6B). The ability of adhesion to polystyrene was calculated through the measurement of total biomass by crystal violent staining in vitro system. After a 2 h, 4 h and 12 h incubation under static condition at 37°C, the total biomass of each sample was examined. Interestingly, the *aft2Δ/Δ* strain had more total biomass in comparison with the wild-type strain, suggesting its stronger adherence ability to polystyrene (Figure 6C). As expected, adhesion phenotypic defect could be rescued by re-expressing *AFT2* fragment under the control of its native promoter. The SEM results also revealed that the number of adhered cells in the *aft2Δ/Δ* mutant was much more than that of the wild-type strain (Figure 6D). Taken together, although the alternations might be attributed to indirect effects caused by *AFT2* deletion, these results still revealed that *C. albicans* Aft2 potentially is responsible for the flocculation, plastic adhesion, and surface hydrophobicity.

### Deletion of *AFT2* Enhances Hyphal Development under Liquid Inducing Conditions

**Figure 7:**
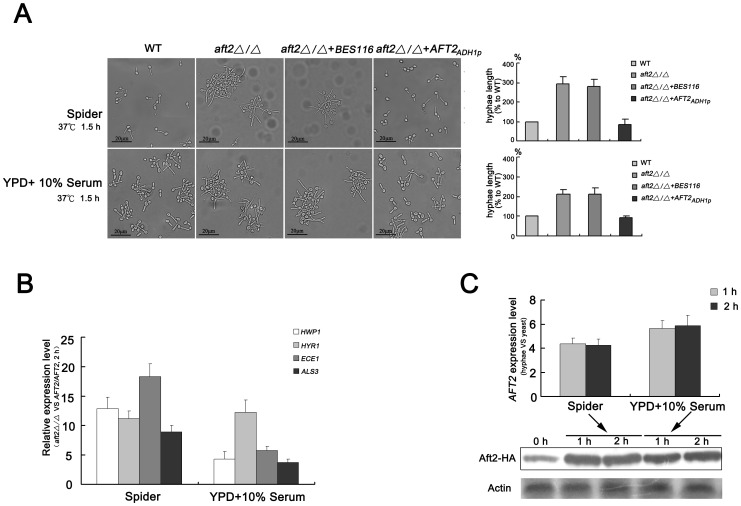
The enhanced hyphal development of *AFT2* deletion is closely associated with increased expression of hypha-specific genes in *C.albicans*. (A) Cells were incubated in liquid YPD+10% serum and Spider medium at 37°C, respectively. At the time indicated, the samples were examined and photographed by light microscopy. Hyphal lengths in the wild-type, *aft2Δ/Δ* mutant, *aft2Δ/Δ*+Vector (NKF90) and *AFT2*-overexpression complemented (NKF91) strains were measured, and shown in the graph. (B) After 2 h incubation in liquid inducing medium, cells were harvested and used for RNA isolation. Quantitative expression levels were determined by real-time PCR assays. (C) The strains producing Aft2-HA fusion proteins were incubated for 0 h, 1 h and 2 h in indicated liquid inducing medium, respectively. Then, cells were harvested and used for RNA isolation and protein extraction. Immunoblot analysis was performed using an anti-HA antibody to detect tagged Aft2 proteins. Actin was detected with anti-actin antibody as a loading control.

To further understand the role of Aft2 in morphogenesis, we examined the phenotypes of the wide-type and *aft2Δ/Δ* mutant strains at 37°C under different liquid hyphal inducing conditions. Interestingly, both in liquid Spider and serum medium, the *aft2Δ/Δ* mutant exhibited an enhanced filamentous aggregation and growth ability in comparison with the wild-type and *AFT2*-overexpression complemented strains (Figure 7A, left). To further understand the role of Aft2 in the formation of hyphae, hyphal lengths in the wild-type and mutant strains were measured. Our data suggested that the hyphal length in the *aft2Δ/Δ* mutant was increased compared with the wild-type and complemented strains (Figure 7A, right). These results supported the conclusion that Aft2 had a role in repressing hyphal development under liquid inducing conditions. To explore the possible mechanism under liquid inducing conditions, quantitative real-time PCR was performed to determine the transcript levels of hypha-specific genes in liquid inducing medium. Consistent with the phenotypes, deletion of *AFT2* in *C. albicans* highly induced the expression of hypha-specific genes, indicating that *C. albicans* Aft2 changed its transcription activity to a repressor under liquid inducing conditions (Figure 7B). In addition, *C. albicans* Aft2 mRNA and protein expression levels were induced under both serum and Spider inducing conditions (Figure 7C). After incubation for 1 h in YPD medium containing 10% serum, the levels of Aft2 mRNA were approximately 5-fold higher than those in YPD medium (marked as 0 h). When the incubation period was extended to 2 h, Aft2 mRNA abundance remained relatively constant in comparison with those for 1 h incubation. Consistent with mRNA levels, the expression levels of Aft2 protein were also elevated during the yeast-to-hypha transition. Similar results were obtained for incubation in liquid Spider medium. In conclusion, our results for the first time demonstrated that *C. albicans* Aft2 has a distinct mechanism in hyphal development by altering its transcription activity under different inducing conditions.

### Iron Deficiency and Environmental Cues Induce Nuclear Import of Aft2 in *C. albicans*

**Figure 8:**
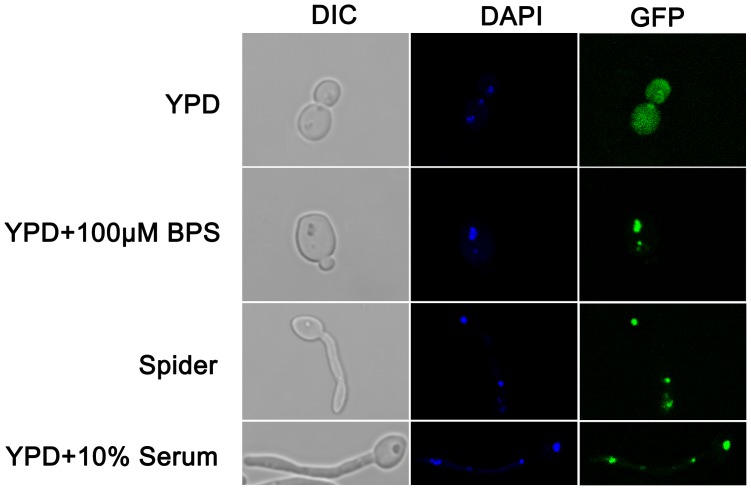
Environmental stimuli induce *C.albicans* Aft2 nuclear accumulation. Overnight cultures of the strain transformed with Aft2-GFP fusion plasmid were regrown to mid-exponential phase in YPD, YPD+100 µM BPS, YPD+10% serum and Spider medium, respectively. Cells were fixed with 3.7% formaldehyde, and then stained with 1 µg/ml 4',6-diamidino-2-phenylindole (DAPI) for 10 min. The subcellular localization of Aft2 was determined by confocal laser scanning microscopy. DIC, Differential interference contrast.

Sequence analyses reveal that *C. albicans* Aft2 harbors two nuclear localization signals in the N-terminal region [31]. To determine whether Aft2 could shuttle between the nucleus and cytoplasm in response to iron levels and morphogenetic signals, we examined the subcellular localization of Aft2 by confocal microscopy. As a control, *C. albicans* strains transformed with empty vector showed no GFP fluorescence signal (data not shown). Our results suggested that Aft2 was localized to the whole cytoplasm in normal condition. However, once iron deficiency occurred, Aft2 shuttled from the cytoplasm into the nucleus, and accumulated in the cell nucleus (Figure 8, second panel). These results verified a hypothesis that iron deficiency induced the accumulation of Aft2 transcription factor in the nucleus, in which the activated Aft2 directly or indirectly regulated the expression of iron-regulon genes in response to iron fluctuations. Likewise, various morphogenetic signals, such as serum and nutrient starvation, affected Aft2 cellular localization. Under hyphal inducing conditions, *C. albicans* Aft2 was also constitutively localized in the nucleus of hyphal cells, indicating the role of Aft2 as a transcription factor in hyphal development (Figure 8, bottom panel). In summary, environmental-induced nucleo-cytoplasmic shuttling of *C. albicans* Aft2 further confirmed our conclusion that Aft2 plays an important role in iron metabolism and hyphal development by regulating the expression of specific genes.

## Discussion

Evolutional analyses of iron-responsive transcriptional regulators reveal that Zn-finger GATA-type transcription repressors are widespread in ascomycete fungi, while Aft-type transcription activators are generally restricted to the *Saccharomycotina* fungi [4], [18], [47], [48]. Iron homeostasis is mainly mediated by Aft-type (*AFT1/AFT2*) positive iron regulatory mechanisms in *S. cerevisiae* [12]. However, GATA-type iron-responsive transcriptional regulator has not been identified in *S. cerevisiae*. Interestingly, numerous iron regulatory pathways have been characterized in *C. albicans*, including Sef1 transcription activator, GATA-type transcription repressor Sfu1 and Hap43-associated CCAAT-binding complex, which are closely associated with the adaptation of iron availability in a hostile environment [17], [18]. Our previous study identified and characterized one Aft-type homologous gene Ca*AFT2*, and found its important role in regulating cell ferric reductase activity in *C. albicans* [31]. Here, we further explored the possible functional roles of *C. albicans* Aft2 in numerous cellular processes.

Deletion of *AFT2* severely decreased whole iron content under iron-deficient conditions, which might be attributed to the reduced expression of iron-regulon genes involved in plasma membrane high-affinity iron uptake system, including the ferrous-specific transport complex composed of a multicopper ferroxidase Fet3 and an iron permease Ftr1. In addition, some members of the *FRE* metalloreductase family, including *FRP1*, *CFL1* and *FRE10*, were also down-regulated in the *aft2Δ/Δ* mutant, indicating a positive regulatory role of Aft2 in activating iron uptake system. On the other hand, *C. albicans* Aft2 could also function as a negative regulator to repress some cellular iron-responsive genes, such as the mitochondrial iron transporter *MRS4*, the vacuolar iron transporter *SMF3*, the siderophore transporter *SIT1* and iron-responsive regulator *HAP43*. In addition, our present studies also revealed that *C. albicans* Aft2 directly regulated *MRS4* expression via the conserved core CACCC site [49]. These results suggested that *C. albicans* Aft2 was closely related to cellular iron mobilization and metabolism. Notably, Hap43 is essential for the growth under iron-limited conditions and virulence of *C. albicans* [19], [20], [32]. Hap43 exerts its functional role in response to iron deprivation by a mechanism involving transcriptional repression of iron utilizing genes, resulting in a transcriptional remodeling shift to iron-independent metabolic pathways. In this study, the *aft2Δ/Δ* mutant displayed a ∼1.9-fold higher expression level of *HAP43* in comparison with the results from wild-type cells, suggesting that *C. albicans* Aft2 might partially regulate iron homeostasis via iron-responsive repressor Hap43 under iron-limited environment. Disruption of *AFT2* resulted in disturbances of iron homeostasis and followed by the activation of iron-responsive regulator Hap43. This process provoked the rearrangement of metabolism and biosynthesis to decrease the utilization of iron-dependent enzymes/pathways, which was believed to facilitate the survival of *C. albicans* in iron-deficient environment. The potential relationship between Aft2 and Hap43 will shed a new insight into the understanding of iron-regulatory mechanisms in *C. albicans*.

The yeast *S. cerevisiae* Aft-type regulator is involved in its oxidative stress resistance. The *aft1Δ*, and particularly *aft1Δaft2Δ*, mutants show hypersensitivity to hydrogen peroxide [13]. H_2_O_2_ has the capacity of causing oxidative damage to cellular components, including DNA, proteins and lipids. Most organisms possess both enzymatic (e.g. superoxide dismutase, catalase, methionine reductase) and non-enzymatic (e.g. glutathione, metallothioneins, glutaredoxin) defense systems to protect cells against oxidative stress [50], [51]. In this study, we demonstrated that *C. albicans* Aft2 was also required for oxidative stress resistance in a concentration-dependent fashion. In addition, we also found that the addition of exogenous iron could not effectively rescue the defective phenotype of the *aft2Δ/Δ* mutant in response to hydrogen peroxide. Further research revealed that the *aft2Δ/Δ* mutant had similar expression levels of iron-regulon genes with the wild-type strain under oxidative stress. Taken together, these data suggested that the role of Aft2 in iron metabolism might be independent of its role in oxidative stress tolerance. Deletion of *AFT2* resulted in hypersensitivity to oxidative stress, which was attributed in part to the increased generation of ROS in the *aft2Δ/Δ* mutant cells. These results also indicated that there was a significant correlation among H_2_O_2_ hypersensitivity of the *aft2Δ/Δ* mutant, the levels of ROS generation and SOD activities. In our model, deletion of *AFT2* triggered a dramatic increase in ROS production in the presence of high H_2_O_2_ concentrations, and subsequently induced the expression levels of SOD activity. The growth defect of the *aft2Δ/Δ* mutant under H_2_O_2_ treatment was caused by increased accumulation of cellular ROS that could not be eliminated efficiently by antioxidant enzymes.

During the long and tedious evolutionary processes, *C. albicans* acquires Aft-type regulatory function before the whole-genome duplication event (WGD), while the *S. cerevisiae* lineage obtains two Aft-type regulators after the WGD [47]. Recent studies reveal that *S. cerevisiae* Aft-type transcription factor Aft1 plays an essential role in remodeling cellular metabolism in addition to iron homeostasis [21], [22], [52]. We have identified a novel Aft-type factor CaAft2 in *C. albicans*, and confirmed that CaAft2 is able to rescue *S. cerevisiae aft1Δ* mutant growth defects under iron-limited conditions [31]. In this study, our results indicated that *C. albicans* Aft2 exhibited an important role in iron metabolism through bi-directional regulation effects on iron-regulon expression, suggesting that the Aft-type regulator in *C. albicans* not only retains but also expands its major conserved function in iron metabolism. However, *C. albicans AFT2* ORF fragment under the control of its native promoter, even in the strong *S. cerevisiae PGK1* promoter, did not effectively restore the *Scaft1Δ* growth defects, suggesting the subdued functional roles of *C. albicans* Aft2 in metal ion sensitivity, alkaline response and other cellular metabolism. The comparison of Aft-type regulator in many cellular processes between *C. albicans* and *S. cerevisiae* provided a potential insight into the evolutional and functional divergence of Aft-type family. In addition, our previous results demonstrated that *C. albicans* Aft2 regulator evolves an important role in morphogenesis and virulence. The ability of adhesion to other cells and surfaces is considered as a remarkable feature of bacteria to offer protection from hostile environment [27], [53], [54]. Adhesion-mediated surface phenotypes are usually marked by cell-cell interactions (flocculation), cell-substrate recognition and adhesion (including plastic adherence and cell surface hydrophobicity) [27]. Cell surface hydrophobicity is considered as an important factor in the ability of adherence to surfaces, which plays a major role in promoting hydrophobic interactions between the cells and abiotic surfaces, and increasing the resistance against the host immune response [27], [55]. Here, we further revealed that *C. albicans* Aft2 was required for invasive growth, flocculation, plastic adhesion, surface hydrophobicity and hyphal development. The *aft2Δ/Δ* mutant exhibited an increased flocculation, a higher surface hydrophobicity and a stronger adherence to abiotic substrates, while the capacity to invade the agar plates was severely attenuated. The inconsistency between invasion and adhesion probably reflected the ability of adhesive specificity towards the appropriate substrates. In contrast to wild-type cells, the *aft2Δ/Δ* cells formed the aberrant and stunted filaments, and failed to penetrate the agar effectively. Additionally, deletion of *AFT2* reduced the expression of hypha-specific genes in solid conditions, including the crucial adhesin *ALS3*. Therefore, the failure of normal filamentous growth and decrease in *ALS3* expression level might be responsible for the attenuated agar invasion.

The ability of the yeast-to-hypha transition is a striking feature of *C. albicans*, which is highly relevant to its pathogenesis and virulence [56]. Though *C. albicans* Aft2 played an important role in colony morphology and hyphal development, there might be two distinct mechanisms by which Aft2 participated in the response to various morphogenetic signals. Our results suggested that *C. albicans* Aft2 functioned as an activator to regulate hypha-specific genes in solid inducing medium, whereas, it switched to a repressor in liquid inducing conditions. Interestingly, *C. albicans* Aft2 mRNA and protein expression levels were also elevated after the incubation in liquid hyphal inducing conditions, suggesting that Aft2-mediated hyphal repression had a lasting effect during the yeast-to-hypha transition. Aft2-mediated hyphal repression could not abolish hyphal development, which might be due to the compromising effects with multiple positive morphogenetic signaling pathways, such as the major morphogenetic processes mediated by Efg1 regulator [24], [57]. In addition, over-expression of *AFT2* under the control of *ADH1* promoter in *aft2Δ/Δ* mutant background could remarkably reduce filamentous aggregation and hyphal elongation, indicating a potential role of Aft2 in repressing hyphal development. Furthermore, environmental cues, including serum and nutrient starvation, affected cellular localization of *C. albicans* Aft2 and its accumulation into the nucleus. The nucleo-cytoplasmic shuttling of *C. albicans* Aft2 was generally considered as a prerequisite to exert its regulatory activity. Thus, it will be an interesting and exciting challenge for future studies to elucidate the mechanisms by which Aft2 functions as both an activator and a repressor, and define its relative position in the network of signal transduction pathway.

## Supporting Information

Figure S1 **Deletion of** ***AFT2*** **has little impact on iron content and iron-regulon expression under iron-adequate conditions.** (A) Overnight cultures of the indicated strains were re-cultivated in 100 ml fresh YPD+200 µM Fe^3+^ medium for 12 h, respectively. Cells were harvested and washed distilled deionized water. Cellular iron content was measured by atomic absorption spectrometry. (B) Overnight cultures of the wild-type and *aft2Δ/Δ* mutant strains were cultivated to mid-exponential phase in YPD+200 µM Fe^3+^ medium, and used for RNA isolation. Quantitative real-time PCR was performed to determine the relative expression changes of iron-responsive genes. Data indicate mean values ± standard deviations from three independent experiments performed in triplicates.
(TIF)

Figure S2 **Quantitative real-time PCR analysis of iron-regulon expression levels in wild type and** ***aft2Δ/Δ*** **mutant cells in response to oxidative stress.** Overnight cultures of the wild-type and *aft2Δ/Δ* mutant strains were cultivated to mid-exponential phase. Then cells were incubated for another 90 min in YPD medium supplemented with 8 mM H_2_O_2_ before harvesting for RNA isolation.
(TIF)
