# Supplementary figures and images for: Aft2, a Novel Transcription Regulator, Is Required for Iron Metabolism, Oxidative Stress, Surface Adhesion and Hyphal Development in Candida albicans
Source: PLoS One. 2013 Apr 23;8(4):e62367. doi: 10.1371/journal.pone.0062367 (PMC3633901; doi:10.1371/journal.pone.0062367)

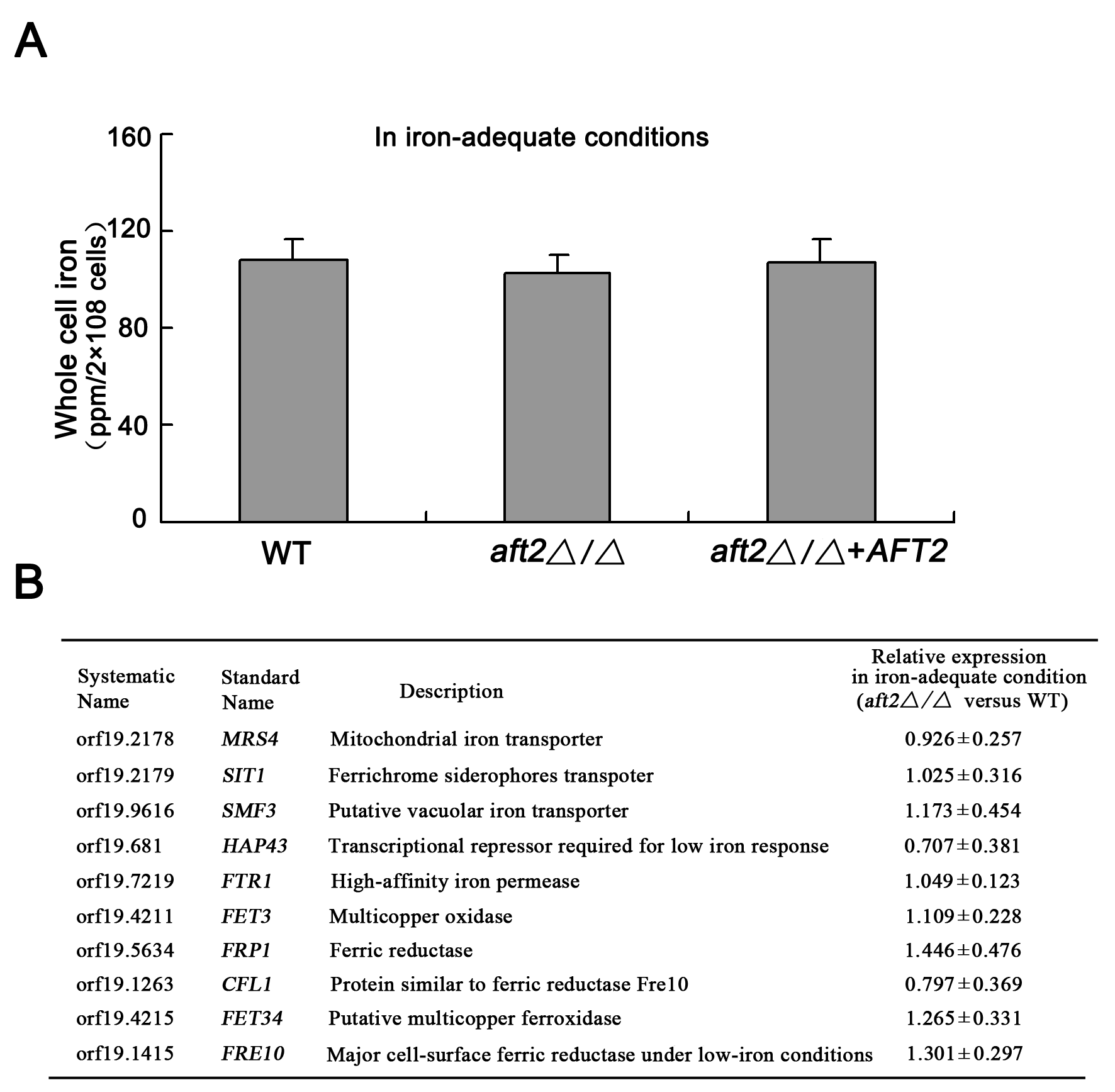

Supplement: Figure S1 — Deletion of AFT2 has little impact on iron content and iron-regulon expression under iron-adequate conditions. (A) Overnight cultures of the indicated strains were re-cultivated in 100 ml fresh YPD+200 µM Fe3+ medium for 12 h, respectively. Cells were harvested and washed distilled deionized water. Cellular iron content was measured by atomic absorption spectrometry. (B) Overnight cultures of the wild-type and aft2Δ/Δ mutant strains were cultivated to mid-exponential phase in YPD+200 µM Fe3+ medium, and used for RNA isolation. Quantitative real-time PCR was performed to determine the relative expression changes of iron-responsive genes. Data indicate mean values ± standard deviations from three independent experiments performed in triplicates. (TIF) [file pone.0062367.s001.tif]

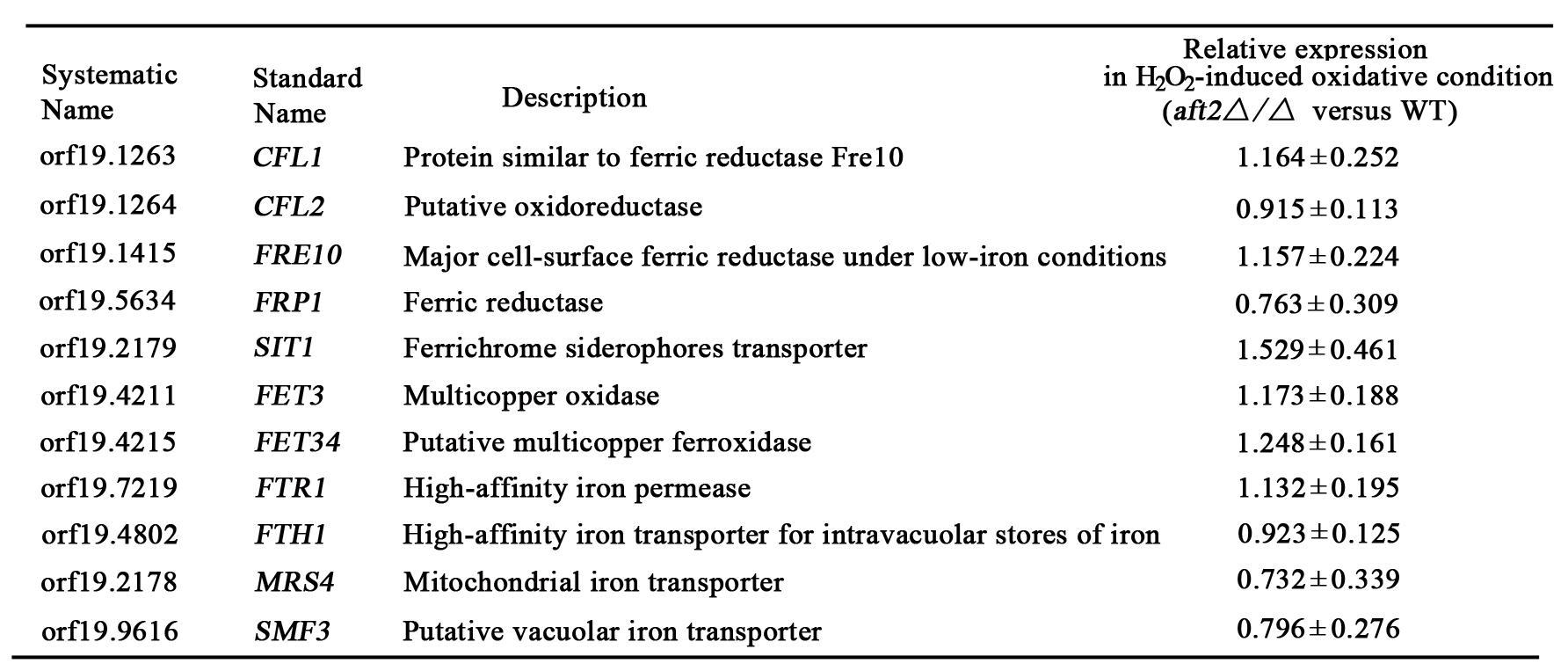

Supplement: Figure S2 — Quantitative real-time PCR analysis of iron-regulon expression levels in wild type and aft2Δ/Δ mutant cells in response to oxidative stress. Overnight cultures of the wild-type and aft2Δ/Δ mutant strains were cultivated to mid-exponential phase. Then cells were incubated for another 90 min in YPD medium supplemented with 8 mM H2O2 before harvesting for RNA isolation. (TIF) [file pone.0062367.s002.tif]
